# Overall survival by clinical risk category for high dose interleukin-2 (HD IL-2) treated patients with metastatic renal cell cancer (mRCC): data from the PROCLAIM^SM^ registry

**DOI:** 10.1186/s40425-019-0567-3

**Published:** 2019-03-27

**Authors:** M. Fishman, J. P. Dutcher, J. I. Clark, A. Alva, G. P. Miletello, B. Curti, Neeraj Agarwal, R. Hauke, K. M. Mahoney, H. Moon, J. Treisman, S. S. Tykodi, G. Daniels, M. A. Morse, M. K. K. Wong, H. Kaufman, N. Gregory, D. F. McDermott

**Affiliations:** 1 Moffitt Cancer Center, Tampa, FL USA; 2 Cancer Research Foundation of NY, Chappaqua, NY USA; 3 Loyola University Medical Center, Maywood, IL USA; 4 University of Michigan, Ann Arbor, MI USA; 5 Hematology/Oncology Clinic, Baton Rouge, LA USA; 6 Earle A. Chiles Research Institute, Providence Cancer Institute, Portland, OR USA; 7 Huntsman Cancer Institute, University of Utah, Salt Lake City, UT USA; 8 Nebraska Cancer Specialist, Omaha, NE USA; 9 Beth Israel Deaconess Medical Center, Boston, MA USA; 10 Southern California Permanente Medical Group, Pasadena, CA USA; 11 Medical College of Wisconsin, Milwaukee, WI USA; 12 University of Washington and Fred Hutchinson Cancer Center, Seattle, WA USA; 13 University of California San Diego, San Diego, CA USA; 14 Duke University, Durham, NC USA; 15 MD Anderson Cancer Center, Houston, TX USA; 16 Massachusetts General Hospital, Boston, MA USA; 17 Prometheus Laboratories, San Diego, CA USA

**Keywords:** Survival, Risk factors, Interleukin-2, Renal cell cancer, PROCLAIM^SM^, Patient registry

## Abstract

**Background:**

Prognostic scoring systems are used to estimate the risk of mortality from metastatic renal cell carcinoma (mRCC). Outcomes from different therapies may vary within each risk group. These survival algorithms have been applied to assess outcomes in patients receiving T-cell checkpoint inhibitory immunotherapy and tyrosine kinase inhibitor therapy, but have not been applied extensively to patients receiving high dose interleukin-2 (HD IL-2) immunotherapy.

**Methods:**

Survival of 810 mRCC patients treated from 2006 to 2017 with high dose IL-2 (aldesleukin) and enrolled in the PROCLAIM^SM^ registry data base was assessed utilizing the International Metastatic RCC Database Consortium (IMDC) risk criteria. Median follow-up is 23.4 months (mo.) (range 0.2–124 mo.). Subgroup evaluations were performed by separating patients by prior or no prior therapy, IL-2 alone, or therapy subsequent to IL-2. Some patients were in two groups. We will focus on the 356 patients who received IL-2 alone, and evaluate outcome by risk factor categories.

**Results:**

Among the 810 patients, 721 were treatment-naïve (89%) and 59% were intermediate risk. Overall, of the 249 patients with favorable risk, the median overall survival (OS) is 63.3 mo. and the 2-year OS is 77.6%. Of 480 patients with intermediate risk, median OS is 42.4 mo., 2-year OS 68.2%, and of 81 patients with poor risk, median OS 14 mo., 2-year OS 40.4%. Among those who received IL-2 alone (356 patients), median OS is 64.5, 57.6, and 14 months for favorable, intermediate and poor risk categories respectively. Two year survival among those treated only with HD IL-2 is 73.4, 63.7 and 39.8%, for favorable, intermediate and poor risk categories respectively.

**Conclusions:**

Among mRCC patients treated with HD IL-2, all risk groups have median and 2-year survival consistent with recent reports of checkpoint or targeted therapies for mRCC. Favorable and intermediate risk (by IMDC) patients treated with HD IL-2 have longer OS compared with poor risk patients, with most durable OS observed in favorable risk patients. Favorable risk patients treated with HD IL-2 alone have a 2-year OS of 74%. These data continue to support a recommendation for HD IL-2 for patients with mRCC who meet eligibility criteria.

**Trial registration:**

PROCLAIM, NCT01415167 was registered with ClinicalTrials.gov on August 11, 2011, and initiated for retrospective data collection until 2006, and prospective data collection ongoing since 2011.

**Electronic supplementary material:**

The online version of this article (10.1186/s40425-019-0567-3) contains supplementary material, which is available to authorized users.

## Background

High dose aldesleukin (HD IL-2), a T-cell growth factor, is an effective immunotherapy for metastatic melanoma (mM) and metastatic renal cell carcinoma (mRCC), yielding a 14–25% objective response rate (ORR) (complete and partial responses) with prolonged response duration, often decades, for complete responders [1–8]. Extensive clinical experience and detailed management guidelines over the last 30 years of HD IL-2 use has ensured predictable short term toxicity and minimal to no lasting residual toxicity [1–9]. As the studies which led to approval for HD IL-2 are decades old, we developed the PROCLAIM^SM^ database, a multi-institutional clinical registry of patients treated with HD IL-2, implemented in 2011, with retrospective data collected back to 2006 and prospective data entered to the present. Seventy-five percent of subjects have been entered prospectively. This is the largest database of real-world outcomes of contemporary IL-2 treatment. Multiple reports have been generated from this database [2, 3, 6–8, 10].

Survival of patients with mRCC is heterogeneous, but may be projected by several prognostic scoring systems based on clinically available risk factors [11–14]. Studies utilizing cytokine therapy identified the following factors as significant for poor survival: elevated lactate dehydrogenase, elevated calcium, anemia with hemoglobin below lower limit of normal (LLN), the renal tumor remaining in place during treatment for metastatic disease, and impaired performance status [11–15]. The International Metastatic Renal Cell Cancer Database Consortium (IMDC) risk criteria have evolved from prior systems [11, 12, 15], and have been successfully applied to assess survival outcomes among patients treated with either anti-vascular endothelial growth factor (VEGF) targeted therapy or immunotherapy [16, 17].

Prognostic scoring systems do not necessarily predict treatment outcome and therefore it is important to assess the efficacy of each new therapy in the different prognostic groups. A recently reported randomized trial evaluated the outcome for patients with mRCC treated with the combination of anti-programmed death − 1 (anti-PD1) and anti-cytotoxic T-lymphocyte-associated antigen-4 (anti-CTLA-4) checkpoint inhibitors (CPI) compared to those treated with sunitinib [18]. Surprisingly, favorable risk patients treated with CPI immunotherapy had a lower 18-month overall survival (OS) compared to favorable risk patients treated with sunitinib, but for patients with intermediate and poor risk, the reverse was observed [18]. In this trial’s analysis, PD-ligand1 (PD-L1) expression was not entirely predictive of CPI benefit, leaving the observed outcome not easily explained. The results of this trial led to FDA approval of the combination of ipilimumab and nivolumab for intermediate and poor risk mRCC patients, but not for those with favorable risk.

In view of the above outcome of more efficacious results with sunitinib compared to CPI immunotherapy in favorable risk mRCC patients, and because patients selected for HD IL-2 treatment meet physiologic parameters allowing them to tolerate treatment and are not selected by IMDC risk criteria specifically [2, 3, 5–10], we have interrogated the PROCLAIM^SM^ HD IL-2 treatment database for survival outcome by IMDC clinical risk criteria of patients with mRCC treated with HD IL-2 [10, 19]. This report represents the first analysis of survival of mRCC patients entered into this database utilizing the IMDC risk factors and risk categories and evaluating their impact on survival.

## Methods

All data in the PROCLAIM database was collected under an ethics committee-approved clinical protocol approved at all contributing clinical sites [19]. Review of patient characteristics finds the registry patients to be consistent with prior reports of HD IL-2 treated patients, with 75% of patients male, median age 57 years, 95% having had nephrectomy, and with 94% specified as having a clear cell component on histology.

The IMDC risk factors for poor survival are the following: a) impaired performance status, b) less than 1 year interval from diagnosis to systemic treatment, c) hemoglobin less than LLN, d) corrected serum calcium above upper LN (ULN), e) neutrophil count above ULN, f) platelet count above ULN. The favorable risk patient group has none of these risk factors; the intermediate risk group has 1–2 risk factors, and the poor risk group has 3 or more risk factors [16, 17].

Among 939 mRCC patients in the database, 810 patients had data for all 6 IMDC criteria. Among the 810 patients, 365 patients had no prior therapy; 414 had therapy following IL-2, 89 had therapy prior to IL-2, and 356 had IL-2 alone, with no prior or subsequent therapy. Some subjects were in more than one group, ie some with no therapy prior, may have had therapy post-IL-2 or IL-2 alone. For 721 patients (89%), HD IL-2 was initial therapy for advanced mRCC.

This report focuses on those who received HD IL-2 alone to assess outcome by risk factor category (Table 1). We provide survival data for those with sequential therapies in Additional file 1: Table S1. Herein we also report response to HD IL-2 and response duration for all patients by risk categories (Table 2).

**Table 1:** Survival by risk groups of 356 patients treated with HD IL-2 alone

	Favorable	Intermediate	Poor
Median OS (months) (95% CI)	64.5 (39.4–112)	57.6 (34.5–62)	14 (4–58)
2 year OS from IL-2 treatment	73.8%	63.7%	39.8%

*OS* overall survival, *95% CI* 95% confidence intervals, *HD* high dose, *IL*-2 Interleukin-2

**Table 2:** Response and clinical benefit by risk category for all patients (*n* = 810)

Best response	Favorable (*n* = 249)	Intermediate (*n* = 480)	Poor risk (*n* = 81)
CR	9 (3.6%)	34 (7%)	1 (1.2%)
PR	52 (21%)	91 (19%)	13 (16%)
SD	128 (51.4%)	185 (38.5%)	24 (30%)
PD	48 (19%)	135 (28%)	34 (42%)
Missing	12 (5%)	35 (7.3%)	9 (11%)
CR + PR	61 (24.5%)	125 (26%)	14 (17.3%)
CR + PR + SD	189 (75.9%)	310 (64.6%)	38 (46.9%)

*CR* complete response, *PR* partial response, *SD* stable disease, *PD* progressive disease

**Fig. 1:**
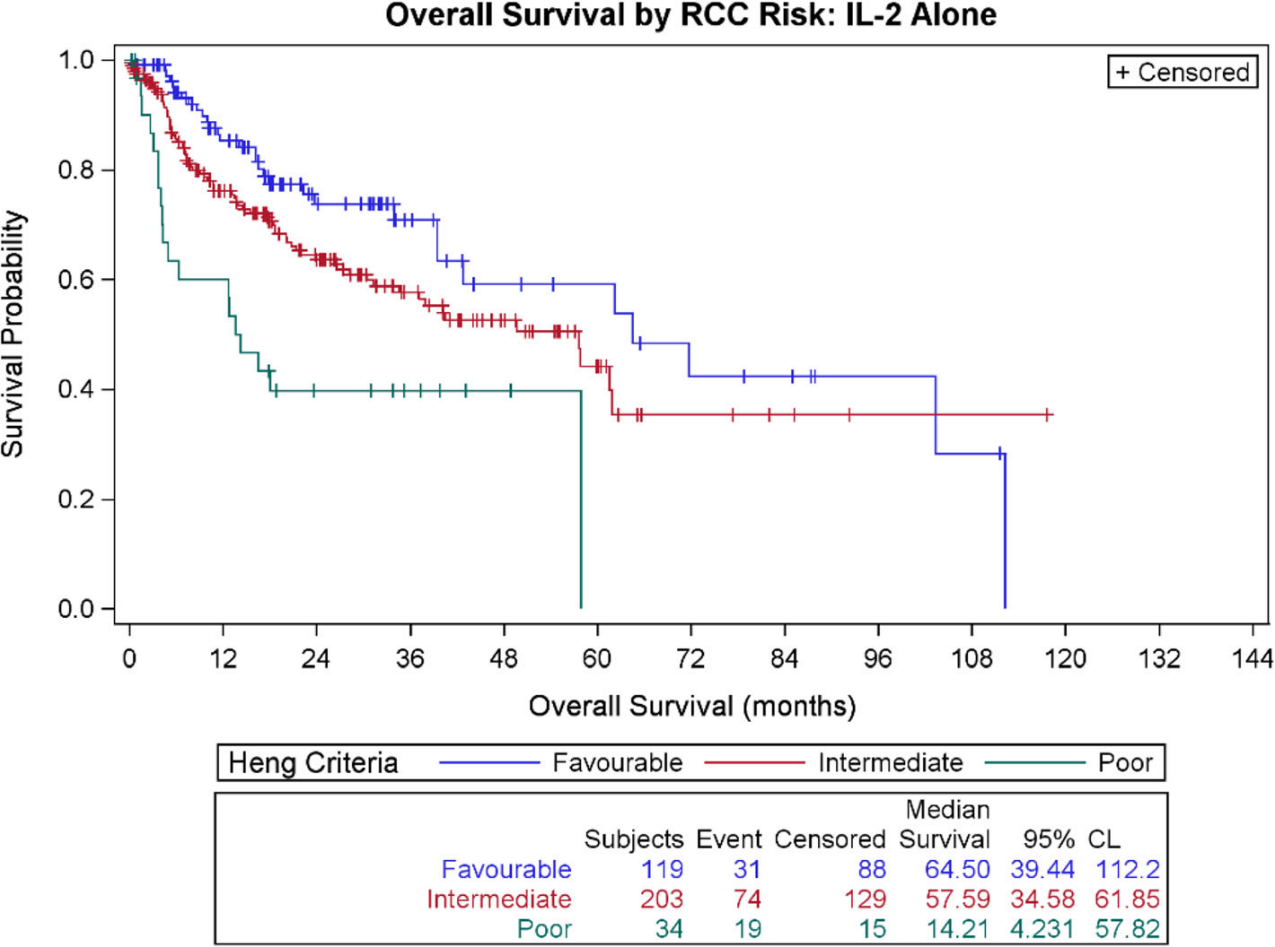
Overall survival by RCC risk: IL-2 alone

Outcome is calculated using product-limit survival estimates by Kaplan-Meier analysis, producing response duration and survival curves. We evaluated the survival outcome of mRCC patients treated with HD IL-2 by IMDC risk category, and by treatment sequence, with OS calculated from initiation of IL-2 treatment in all groups. Figure 1 presents data for those treated with IL-2 alone by risk group. Additional file 1: Figure S1 presents data for all 810 patients and Additional file 1: Figure S2 presents survival for those who received therapy post IL-2. Additional file 1: Figures S3–S5 demonstrate complete and partial response and stable disease duration for all responders by risk category. Additional file 1: Figure S6 presents OS for the small group with therapy prior to IL-2, calculated from the start of the initial therapy for mRCC.

## Results

Among the 810 patients, approximately 25% were entered retrospectively, and 75% were entered prospectively. The median follow-up is 23.4 months (range 0.2–124+ months). Overall, 721 patients (89%) in this registry cohort were treatment-naїve prior to receiving IL-2 and were in the intermediate risk category (59%).

### Survival for patients treated with HD IL-2 alone

Among the 356 patients treated with HD IL-2 alone, 119 met favorable, 203 (57%) met intermediate and 34 met poor risk criteria. This distribution is characteristic of mRCC patients undergoing HD IL-2 or other systemic treatment, in that more than 50% of mRCC patients undergoing initial treatment for advanced disease are in the intermediate risk category. Clinical factors delineating eligibility for IL-2 therapy may somewhat increase the proportion of favorable risk patients, however.

The median OS for favorable, intermediate and poor risk groups treated with IL-2 alone is 64.5 months, 57.6 months, and 14 months, respectively (Table 1). The 2-year OS for those treated with IL-2 alone by risk category is 73.8, 63.7, and 39.8% respectively (Table 1). Figure 1 presents the Kaplan-Meier OS curves for patients treated with IL-2 alone by risk categories.

Thus, the median OS observed among the favorable risk patients is followed closely by the median OS for those in the intermediate risk group (only 6 months difference between favorable and intermediate risk groups for those receiving IL-2 alone). The favorable risk group has a median survival greater than 5 years, and the intermediate risk patients have a median survival 2 months less than 5 years following IL-2 alone.

### Response to HD IL-2

Among all patients with all 6 IMDC criteria known (*n* = 810), 44 achieved complete response (CR) (5.4%), 156 achieved partial response (PR) (19%), and 337 achieved stable disease (SD) (41.6%) as best response (Table 2). Median response duration for all CR and PR patients in favorable and intermediate risk groups was >/= 5+ years (Additional file 1: Figures S3–S5), which is consistent with clinical trial data demonstrating durable CR and PR results following treatment with HD IL-2 [5]. Favorable risk patients achieving SD following IL-2 had median duration of SD of 49 months and intermediate risk SD patients had median duration of SD of 24 months.

### Supplemental survival data

Survival data for all 810 IL-2 treated patients and for the subgroups who received therapy before or after HD IL-2 are presented in the supplemental file. Median OS and 2-year OS by risk group and treatment sequence are presented in Additional file 1: Table S1a and b. Additional file 1: Figure S1 shows OS by risk category for all patients. For the 249 patients with favorable risk, the median OS is 63.3 months and the 2-year OS is 77.6%, and these results are similar when broken down by the treatment groups. For the 480 patients with intermediate risk, median OS is 43.4 months and 2-year OS is 68.2%. Among the 81 patients with poor risk, median OS is 14 months and 2-year OS is 40.4%.

Patients treated with additional therapy following progression after HD IL-2 appeared to benefit from follow-on therapy, and our data suggest enhanced 2-year OS in this group, whether treated with subsequent anti-VEGF targeted therapy (more than 80% of subsequent treatment) or immunotherapy (Additional file 1: Table S1 and Figure S2). This is consistent with a previous report in smaller numbers of patients that evaluated post-IL-2 targeted therapy in patients with mRCC [6]. This could reflect a) efficacy of the subsequent therapy independent of IL-2, b) the physiologic condition of patients eligible to receive HD IL-2 and subsequent therapy, and/or c) a biological interaction between prior IL-2 therapy and ability to benefit from subsequent therapies. Again, the limitations of registry data apply to any interpretation of these data. Additionally, in this registry cohort, only 89 patients received therapy prior to IL-2, again, predominantly anti-VEGF targeted therapy.

## Discussion

In this analysis of mRCC patients in the PROCLAIM^SM^ HD IL-2 registry, all IMDC risk groups have median and 2-year survivals following HD IL-2 that are consistent with recent reports of CPI immunotherapy or anti-VEGF targeted therapy for mRCC. All risk categories have improved survival compared with historical cytokine data [11–15] and are consistent with data from a recent prospective study, IL-2 “Select” [5] and other contemporary reports [2–4, 6–8].

In the high dose IL-2 alone patient group, favorable and intermediate risk patients demonstrate prolonged OS, and many experience years of treatment-free survival following IL-2 therapy. Further, the prolonged OS and 74% 2-year survival for the favorable risk group treated with IL-2 alone is in contrast to the outcome from the randomized trial of combined checkpoint inhibition, in which the favorable risk group had a better 18-mo OS with sunitinib compared with CPI [18]. This contrast demonstrates the nuances and differences between different categories of immunotherapy in the treatment of mRCC that are yet to be sorted out.

Eligibility for HD IL-2 treatment includes physiologic evaluation which may enrich for favorable risk patients, although the percentage of intermediate risk patients is similar to other mRCC treatment reports. Nevertheless, these eligibility criteria might inadvertently select an even better subset of each IMDC risk group for outcome when treated with IL-2 alone or with IL-2 followed by subsequent therapy, yielding durable response and survival. In support of this concept, even poor risk patients meeting IL-2 therapy criteria demonstrated clinical benefit with best clinical responses of CR, PR and SD reported, and 2 year OS of 40–50%.

Among responders in all treatment sequences, median response duration (CR and PR) following HD IL-2 was greater than or equal to 5 years regardless of risk category. However, durable stable disease was observed primarily in favorable risk (median > 5 years) and intermediate risk (median > 3 years) groups, with 10% durable SD among the poor risk group.

The CR rate of 5.4% is consistent with prior reports of IL-2 alone, but less than that of the combination ipilimumab/nivolumab arm of Checkpoint 214 in first line patients [18]. However, it is of note that the CR rate for single agent nivolumab in mRCC in both a large clinical trial experience and a large registry experience is 1 and 1.2%, respectively [20, 21]. Additionally, the CR plus PR rate for HD IL-2 alone is 25%, again consistent with prior reports of IL-2 and possibly allowing for resection of residual disease in PR patients, yielding “surgical CRs”, often with long-term disease-free intervals. The CR + PR rate for nivolumab alone was reported as 25 and 20% [20, 21].

### Limitations of registry data

Therapy-specific registries have helped provide real-world data and increased safety data on therapies beyond the initial clinical trials leading to drug approvals, and thus provide more insight into the spectrum of use of therapies. Examples are the International Bone Marrow Transplant Registry (IBMTR), the IMDC, and the initial registries of patients treated with anti-VEGF targeted therapies for mRCC patients off protocol, following regulatory approvals, but before general availability of these medications. The PROCLAIM registry has provided insight into optimization of IL-2 management and patient selection for this therapy with a goal of maximizing benefit from IL-2 [19, 22].

With decades of experience, careful patient selection and explicit treatment eligibility criteria have enhanced the safety and efficacy of IL-2, but such selection may limit generalizability of conclusions. There are limitations to registry data in that it may not be audited or reviewed, relying on investigator reporting, and there are limitations to the amount of data collectable from sites, as well as variations in documentation, which may limit comparability. Additionally, patients may not be enrolled in a consecutive fashion, depending on other treatment options or choices available at various sites.

### Combination of HD IL-2 with checkpoint inhibitors, sequential and concurrent treatment

In view of the well-known durability of response (DOR) to HD IL-2 from many reports, and the observation of durable survival of mRCC patients following HD IL-2, particularly in favorable and intermediate risk groups in this report, combination therapy, both in sequence and concurrently, to enhance the complete response rate, DOR and OS is being investigated. Metastatic RCC responders to HD IL-2 have among the most durable survival data of mRCC patients with any treatment [2–8, 10, 12, 16–18]. A series of recent reports suggest the feasibility of combinations, either sequentially or concurrently.

Safety and efficacy of HD IL-2 followed by anti-VEGF targeted therapies for stable or progressive disease has been previously reported from the PROCLAIM registry data, and confirmed in this larger cohort (Additional file 1: Table S1a, b), demonstrating enhanced OS [6]. The increased use of anti-VEGF therapy and CPI therapy as initial treatment has also led to further evaluation of sequence, with these therapies preceding HD IL-2. As presented in the supplemental data patients eligible to receive HD IL-2 following progression on anti-VEGF therapy have outcomes similar to those receiving HD IL-2 alone (Additional file 1: Table S1b).

Buchbinder et al. has published retrospective data demonstrating activity and no additive or unexpected toxicity among melanoma patients treated with HD IL-2 following progression after ipilimumab (Ipi) [23]. More recently, they reported safety and efficacy of HD IL-2 treatment in patients who had previously received anti-PD1 therapy and then progressed, (including both melanoma and mRCC patients) with outcomes similar to patients treated with IL-2 alone as first line therapy [24]. HD IL-2 was both active and safe in patients who had no ongoing immune-related adverse events (iRAEs) other than hypothyroid disease undergoing replacement therapy [24]. A prospective evaluation of this sequence is planned.

With respect to concurrent administration of HD IL-2 and CPI therapy, early feasibility was reported by Prieto et al. in an initial clinical study report and a 7-year follow-up of 36 melanoma patients treated with the combination of Ipi and HD IL-2 [25, 26]. The schedule was dose 1 of Ipi given alone, followed by dosing every 3 weeks, as tolerated, with the combination of one dose of Ipi and then HD IL-2 720,000 units per kilogram (U/kg) every 8 h up to 15 doses, with the first dose of IL-2 given within 24 h of the Ipi dose. Although initially a dose-finding study, 24 of 36 patients were treated at 3 mg/kg of Ipi. The CR rate (calculated among all 36 patients) was 17%, with all CR’s ongoing at the time of the long-term follow-up report and the longest duration being 89+ months in that report [26]. Of interest, grade III/IV iRAEs were 17% among the Ipi/IL-2 patients, in contrast to grade III/IV iRAE rates of 29 and 32% in two parallel Ipi/vaccine trials conducted at the same doses and schedules of Ipi by this group [25, 26]. Also of interest, among the Ipi/IL-2 patients, there was no correlation between response and development of iRAEs, but the numbers are small. The high CR rate and tolerable toxicity as well as the durability of responses observed, strongly suggest that further combination studies of HD IL-2 and CPIs should be evaluated, for the potential of enhancing CR rate, potentially leading to durable responses and enhanced 2 year and overall survival.

Several ongoing investigator initiated trials are evaluating concurrent or rapid sequential use of HD IL-2 and anti-PD1 agents, in both melanoma and mRCC, and are evaluating immune parameters and biomarkers (Additional file 1: Table S2). These studies are ongoing, several are dose-finding for IL-2, and to date no unusual safety signals have been reported. The clinical trial that is furthest along is NCT02964078, in mRCC patients, with concurrent pembrolizumab and IL-2, 600,000 U/kg, but utilizing a different IL-2 schedule, with 5 doses administered over 33 h, similar to a previously published regimen [27]. Preliminary data were reported at the February 2019 annual meeting of the Genitourinary American Society of Clinical Oncology (ASCO) and describe a higher than additive response rate with no prohibitive toxicity [28]. Follow-up is ongoing.

## Conclusions

HD IL-2 treatment yields durable responses among mRCC patients of all risk categories eligible for HD IL-2 therapy, and prolonged survival among mRCC patients, particularly in the favorable and intermediate risk groups. This therapy has the advantage of yielding prolonged treatment-free survival among responders. Additionally, data supporting feasibility of concurrent administration of IL-2 with CPIs is accumulating, potentially enhancing response rates. HD IL-2 remains an important treatment option for mRCC patients meeting eligibility criteria, both as first line and subsequent therapy. Combination studies are ongoing.

## Additional file

Additional file 1: **Table** **S1a.** Median survival (months) by risk group and therapy sequence. **Table S1b.** Two year overall survival by risk group and treatment sequence. **Table S2.** Ongoing trials of IL-2 and checkpoint inhibitors. **Figure S1.** Overall survival by RCC risk: all patients with 6 IMDC criteria. **Figure S2.** Overall survival by RCC risk: post-IL-2 treatment. **Figure S3.** Response duration: all complete response patients. **Figure S4.** Response duration: All partial response patients. **Figure S5.** Response duration: all stable disease patients. **Figure S6.** Overall survival by RCC risk: treatment prior to IL-2 from first treatment date. (DOCX 549 kb)

## Abbreviations

ASCO: American Society of Clinical Oncology
CR: Complete response
CTLA4: Cytotoxic T-lymphocyte-associated antgen-4
FDA: Food and Drug Administration
HD IL-2: High dose interleukin-2
IBMTR: International bone marrow transplant registry
IMDC: International metastatic renal cell cancer database consortium
IPI: Ipilimumab
iRAE: Immune-related adverse event
LLN: Lower limit of normal
Mo.: Months
mRCC: Metastatic renal cell carcinoma
ORR: Objective response rate
OS: Overall survival
PD1: Programmed death 1
PR: Partial response
SD: Stable disease
U/kg: Units per kilogram
ULN: Upper limit of normal
VEGF: Vascular endothelial growth factor

## References

[1] Fisher RI, Rosenberg SA, Fyfe G (2000). Long-term survival update for high-dose recombinant interleukin-2 in patients with renal cell carcinoma. Cancer J Sci Amer.

[2] Clark JI, Morse MA, Wong MKK, McDermott DF, Kaufman HL, Daniels GA (2015). Durability of responses in patients with metastatic renal cell carcinoma treated with HD IL-2. JITC.

[3] Alva A, Daniels GA, Wong MK, Kaufman H, Morse MA, McDermott DF (2016). Contemporary experience with high-dose interleukin-2 therapy and impact on survival in patients with metastatic melanoma and metastatic renal cell carcinoma. Cancer Immunol Immunother.

[4] Stenehjem DD, Toole M, Merriman J, Parikh K, Daignault S, Scarlett S (2016). Extension of overall survival beyond objective responses in patients with metastatic renal cell carcinoma treated with high-dose interleukin-2. Cancer Immunol Immunother.

[5] McDermott DF, Cheng SC, Signoretti S, Margolin KA, Clark JI, Sosman JA (2015). The high-dose aldesleukin “select” trial: a trial to prospectively validate predictive models of response to treatment in patients with metastatic renal cell carcinoma. Clin Cancer Res.

[6] Clark JI, Wong MKK, Kaufman HL, Daniels GA, Morse MA, McDermott DF (2017). Impact of sequencing targeted therapies with high-dose interleukin-2 immunotherapy: an analysis of outcome and survival of patients with metastatic renal cell carcinoma from an on-going observational IL-2 clinical trial. PROCLAIMSM. Clin Genitourin Cancer.

[7] Clark JI, Curti B, Davis E, Kaufman H, Amin A, Alva A (2017). Long-term disease-free survival of melanoma and renal cell cancer patients following high-dose interleukin-2. JITC.

[8] Curti B, Daniels GA, McDermott DF, Clark JI, Kaufman HL, Logan TF (2017). Improved survival and tumor control with Interleukin-2 is associated with the development of immune-related adverse events: data from the PROCLAIM^SM^ registry. JITC.

[9] Dutcher JP, Schwartzentruber DJ, Kaufman HL, Agarwala SS, Tarhini AA, Lowder J, Atkins MB (2014). High dose Interleukin-2 (Aldesleukin) – expert consensus on best management practices – 2014. JITC.

[10] Fishman M, Clark JI, Alva A, Curti B, Agarwal N, Hauke R (2018). Overall survival by clinical risk category for high dose Interleukin-2 treated metastatic renal cell cancer: data from PROCLAIM^SM^. J Clin Oncol.

[11] Motzer RJ, Mazumdar M, Bacik J, Berg W, Amsterdam A, Ferrara J (1999). Survival and prognostic stratification of 670 patients with advanced renal cell carcinoma. J Clin Oncol.

[12] Motzer RJ, Mazumdar M, Bacik J, Russo P, Berg WJ, Metz E (2000). Effect of cytokine therapy on survival for patients with advanced renal cell carcinoma. J Clin Oncol.

[13] Manola J, Royston P, Elson P, McCormack JB, Mazumdar M, Negrier S (2011). Prognostic model for survival in patients with metastatic renal cell carcinoma: results from the international kidney cancer working group. Clin Cancer Res.

[14] Palmer PA, Vinke J, Philip T, Negrier S, Atzpodien J, Kirchner H (1992). Prognostic factors for survival in patients with advanced renal cell carcinoma treated with recombinant interleukin-2. Ann Oncol.

[15] Negrier S, Escudier B, Gomez F, Douillard JY, Ravaud A, Chevreau C (2002). Prognostic factors of survival and rapid progression in 782 patients with metastatic renal carcinomas treated by cytokines: a report from the Groupe Français d’Immunothérapie. Ann Oncol.

[16] Heng DY, Xie W, Regan MM, Warren MA, Golshayan AR, Sahi C (2009). Prognostic factors for overall survival in patients with metastatic renal cell carcinoma treated with vascular endothelial growth factor-targeted agents: results from a large, multicenter study. J Clin Oncol.

[17] Heng DY, Xie W, Regan MM, Harshman LC, Bjarnason GA, Vaishampayan UN (2013). External validation and comparison with other models of the international metastatic renal –cell carcinoma database consortium prognostic model: a population-based study. Lancet Oncol.

[18] Motzer RJ, Tannir NM, McDermott DF, Aren-Frontera O, Melichar B, Choueiri TK (2018). Nivolumab plus ipilimumab versus sunitinib in advanced renal-cell carcinoma. N Engl J Med.

[19] Kaufman HL, Wong MK, Daniels GA, McDermott DF, Aung S, Lowder J, Morse MA (2014). The use of registries to improve cancer treatment: a national database for patients treated with interleukin-2 (IL-2). J Personalized Med.

[20] Motzer RJ, Escudier B, McDermott DF, George S, Hammers HJ, Srinivas S (2015). Nivolumab versus everolimus in advanced renal cell carcinoma. N Engl J Med.

[21] Albiges L, Negrier S, Dalban C, Chevreau C, Gravis G, Oudard S (2019). Safety and efficacy of nivolumab in metastatic renal cell carcinoma (mRCC): final analysis from NIVOREN GETUG AFU 26 study. J Clin Oncol.

[22] Wong MK, Kaufman HL, Daniels GA, McDermott DF, Aung S, Lowder JN, Morse MA (2014). Commentary: Implementation of an interleukin-2 national registry: an opportunity to improve cancer outcomes. JITC.

[23] Buchbinder EI, Gunturi A, Perritt J, Dutcher J, Aung S, Kaufman HL (2016). A retrospective analysis of high dose interleukin-2 following ipilimumab in metastatic melanoma. JITC.

[24] Buchbinder EI, Dutcher JP, Daniels GA, Curti BD, Patel SP, Holtan SG (2019). Therapy with high-dose interleukin-2 in metastatic melanoma and renal cell carcinoma following PD1 or PDL1 inhibition. JITC.

[25] Maker AV, Phan GQ, Attia P, Yang JC, Sherry RM, Topalian SL (2005). Tumor regression and autoimmunity in patients treated with cytotoxic T lymphocyte-associated antigen 4 blockade and interleukin-2: a phase I/II study. Ann Surg Oncol.

[26] Prieto PA, Yang JC, Sherry RM, Hughes MS, Kammula US, White DE (2012). CTLA-4 blockade with ipilimumab: long-term follow-up of 177 patients with metastatic melanoma. Clin Cancer Res.

[27] Finkelstein SE, Carey T, Fricke I, Daohai Y, Goetz D, Gratz M (2010). Changes in dendritic cell phenotype after a new high-dose weekly schedule of interleukin-2 therapy for kidney cancer and melanoma. J Immunother.

[28] Chatzkel J, Swank J, Ludlow S, Lombardi K, Croft C, Artigas Y (2019). Overall responses with coordinated pembrolizumab and high dose IL-2 (5-in-a-row schedule) for therapy of metastatic clear cell renal cancer, a single center, single arm trial. J Clin Oncol.

