# Supplementary material for: Overall survival by clinical risk category for high dose interleukin-2 (HD IL-2) treated patients with metastatic renal cell cancer (mRCC): data from the PROCLAIMSM registry
Source: J Immunother Cancer. 2019 Mar 27;7:84. doi: 10.1186/s40425-019-0567-3 (PMC6437874; doi:10.1186/s40425-019-0567-3)

**JITC-D-18-00507**

**OVERALL SURVIVAL BY CLINICAL RISK CATEGORY FOR HIGH DOSE INTERLEUKIN-2 (HD IL-2) TREATED PATIENTS WITH METASTATIC RENAL CELL CANCER (mRCC): DATA FROM THE PROCLAIM^SM^ REGISTRY**

**M Fishman^1^, JP Dutcher ^2^, JI Clark^3^, A Alva^4^, GP Miletello^5^, B Curti^6^, Neeraj Agarwal^7^, R Hauke^8^, KM Mahoney^9^, H Moon^10^, J Treisman^11^, SS Tykodi^12^, G Daniels^13^, MA Morse^14^, MKK Wong^15^, H Kaufman^16^, N Gregory^17^, DF McDermott^9^**

**SUPPLEMENTAL MATERIALS**

Supplemental Table S1a: Median Survival (months) by Risk Group and Therapy Sequence

Supplemental Table S1b: Two Year Overall Survival by Risk Group and Treatment Sequence

Supplemental Table S2: Ongoing Trials of IL-2 and Checkpoint Inhibitors

**SUPPLEMENTAL FIGURE LEGENDS**

Figure S1: Overall Survival by RCC Risk: All Patients with 6 IMDC Criteria.

Figure S2: Overall Survival by RCC Risk: Post-IL-2 Treatment

Figure S3: Response Duration: All Complete Response Patients

Figure S4: Response Duration: All Partial Response Patients

Figure S5: Response Duration: All Stable Disease Patients

Figure S6: Overall Survival by RCC Risk: Treatment Prior to IL-2 from First Treatment Date

**Table S1a: Median Survival (months) by Risk Group and Therapy Sequence**

Patient Group Favorable Intermediate Poor Risk
 Median Survival Median Survival Median Survival
 (95% CI) (95% CI) (95% CI)

| All Patients with 6 IMDC criteria  (n=810) | 63.3 mo  (51.2-73) | 42.4 mo  (35.4-56) | 14 mo  (10-24.5) |
| --- | --- | --- | --- |
| No Therapy Prior to IL-2 (n= 365) | 60 mo  (51-78) | 41 mo  (33.5-51) | 24.5 mo  (10-30) |
| Therapy post IL-2  (n= 414) | 60 mo  (51-78) | 41.6 mo  (33.5-51) | 22.5 mo (10-27.5) |
| IL-2 Alone  (n= 356) | 64.5 mo  (39.4-112) | 57.6 mo  (34.5-62) | 14 mo  (4-58) |

95% CI-- 95% confidence intervals

**Table S1b: Two Year Overall Survival by Risk Group and Treatment Sequence**

Patient Group Favorable Intermediate Poor Risk Total

| All patients  (n=810) | 77.6% | 68.2% | 40.4% | 68.2% |
| --- | --- | --- | --- | --- |
| No therapy prior to IL-2  (n=365) | 83.6% | 71.6% | 53.3% | 73.9% |
| Therapy Prior to IL-2  (n=89) | 70.6% | 50.9% | 93.3% | 61.8% |
| Therapy Post  IL-2  (n=414) | 81.0% | 72.0% | 46.8% | 72.4% |
| IL-2 Alone  (n=356) | 73.8% | 63.7% | 39.8% | 64.5% |

**Table S2: Ongoing Trials of IL-2 and Checkpoint Inhibitors**

| **mRCC** |  |  |
| --- | --- | --- |
| NCT02964078 | 4 Blocks of 9 wks each:  Pembro wk 1,4,7  HD IL-2, 5 doses over 33 hrs, wks 2,3, 5,6 starting second 9 wk block | Ref 28 |
| NCT02989714 | Wk 1, HD IL-2, cycle 1  Wk 2, Nivo  Wk 3, HD IL-2, cycle 2  Wk 4 and every 2 wks, Nivo |  |
| NCT03111901 | Cycle 1: LD IL-2, Day 1-5, Day 8-12; Pembro D2  Cycle 2 and beyond up to 2 years:  Pembro d 1 every 21 days; LD IL-2, D 1-5, D8-12 |  |
| NCT03260504 | Pembro, wk 1 and every 3 weeks;  IL-2 – Cohort 1, SQ low dose wks 1-6;  IL-2 – Cohort 2, low dose IV, wks 1 and 4  IL-2 – Cohort 3, HD IL-2, IV wks 1 and 4 |  |
| **mMelanoma** |  |  |
| NCT02748564 | Pembro, wk 0 and every 3 wks;  IL-2 – Cohort 1, LD IV, wk 3 and 5  IL-2 – Cohort 2, Int Dose IV, wk 3 and 5  IL-2 - Cohort 3, HD IL-2 IV, wk 3 and 5 |  |
| NCT03476174 | Pembro every 3 wks x 2 doses  HD IL-2 – 2 cycles |  |
| NCT03111901 | As above |  |

**SUPPLEMENTAL FIGURES**

Figure S1: Overall Survival by RCC Risk: All Patients


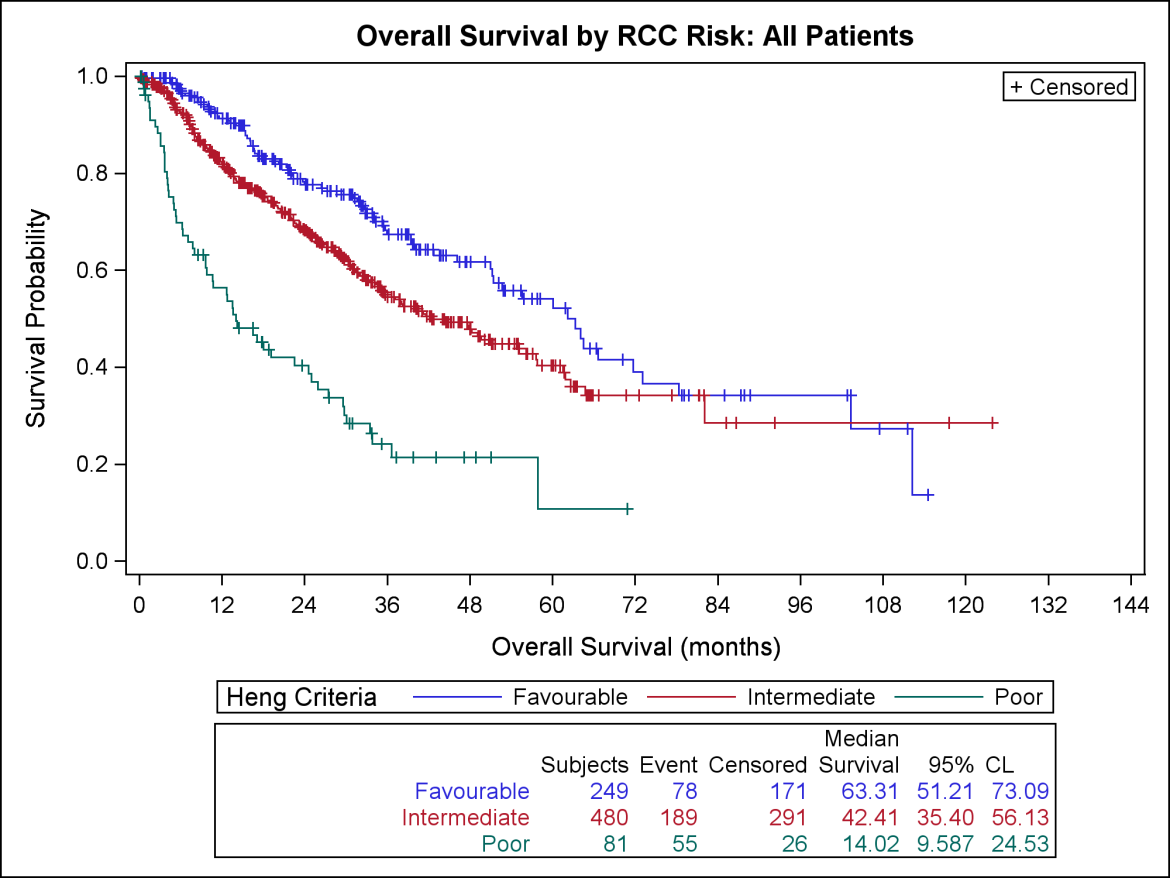


Figure S2: Overall Survival by RCC Risk: Post IL-2 Treatment


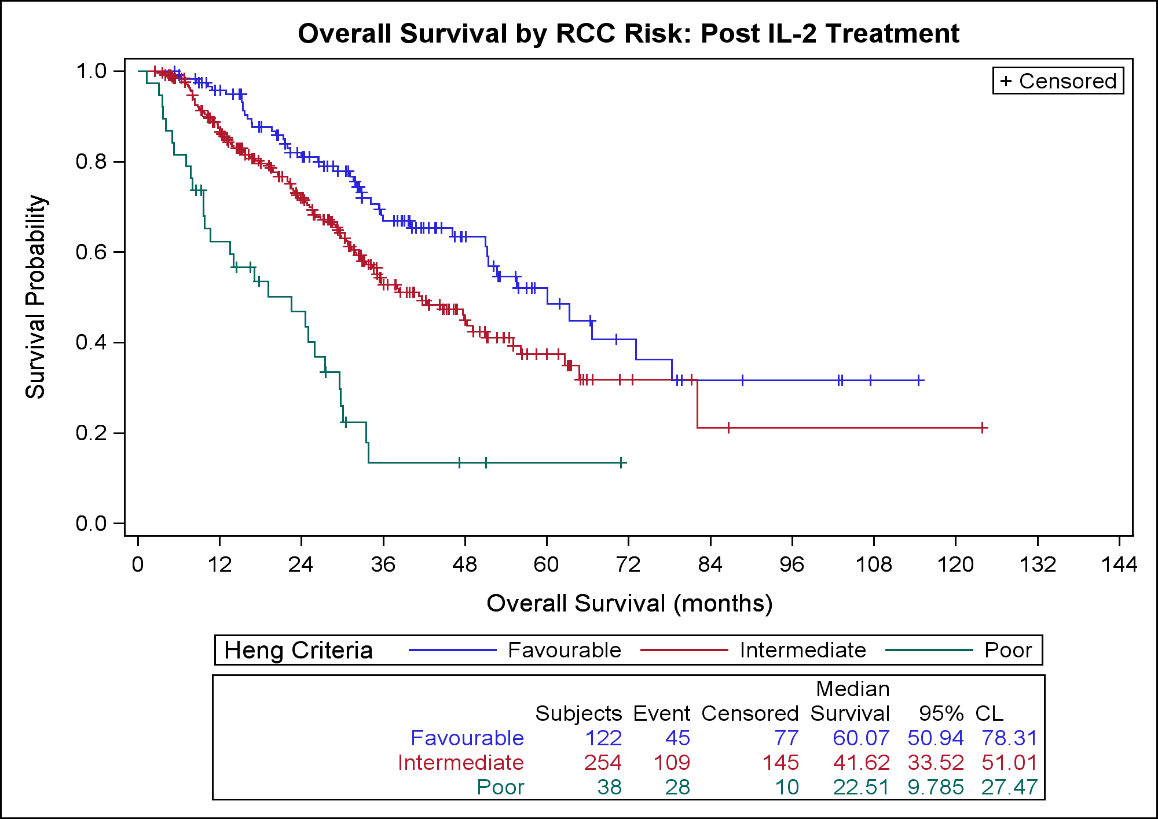


Figure S3: Response Duration: All Complete Response Patients


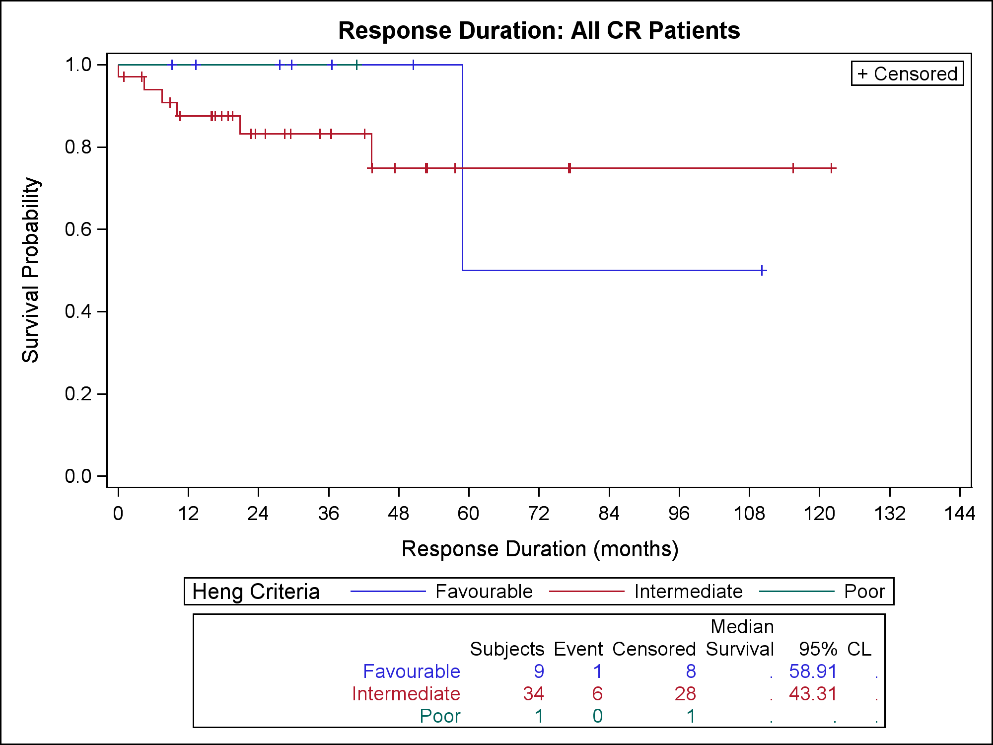


Figure S4: Response Duration: All Partial Response Patients


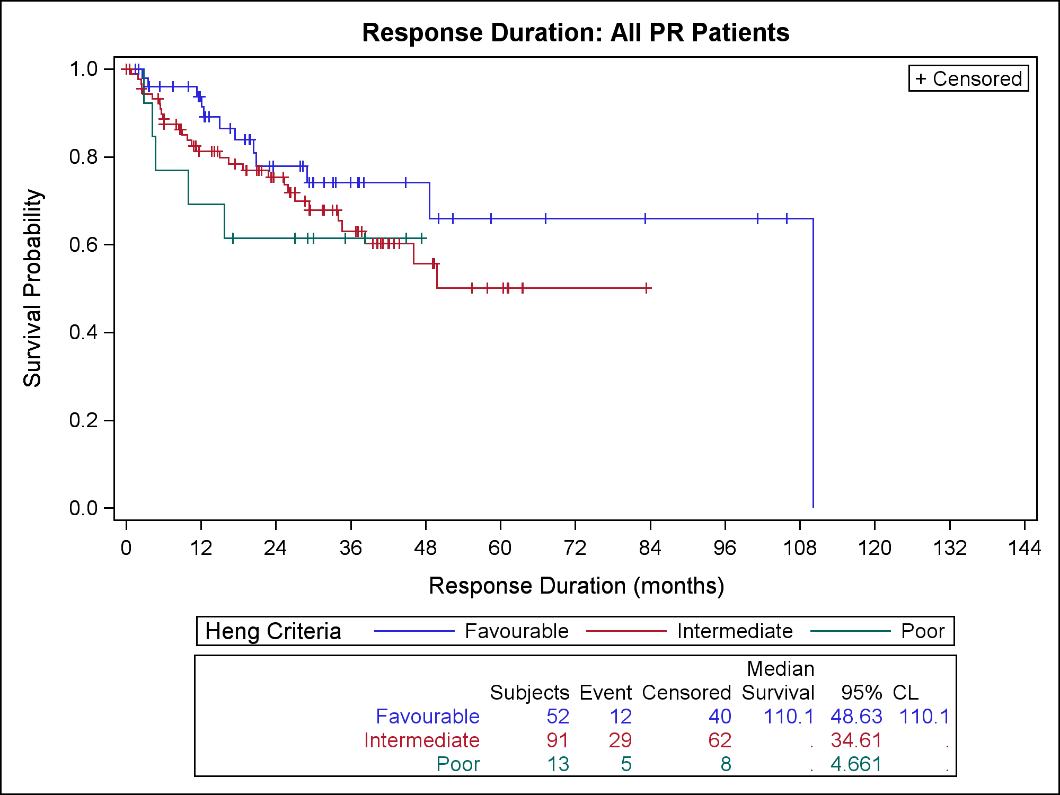


Figure S5: Response Duration: All Stable Disease Patients


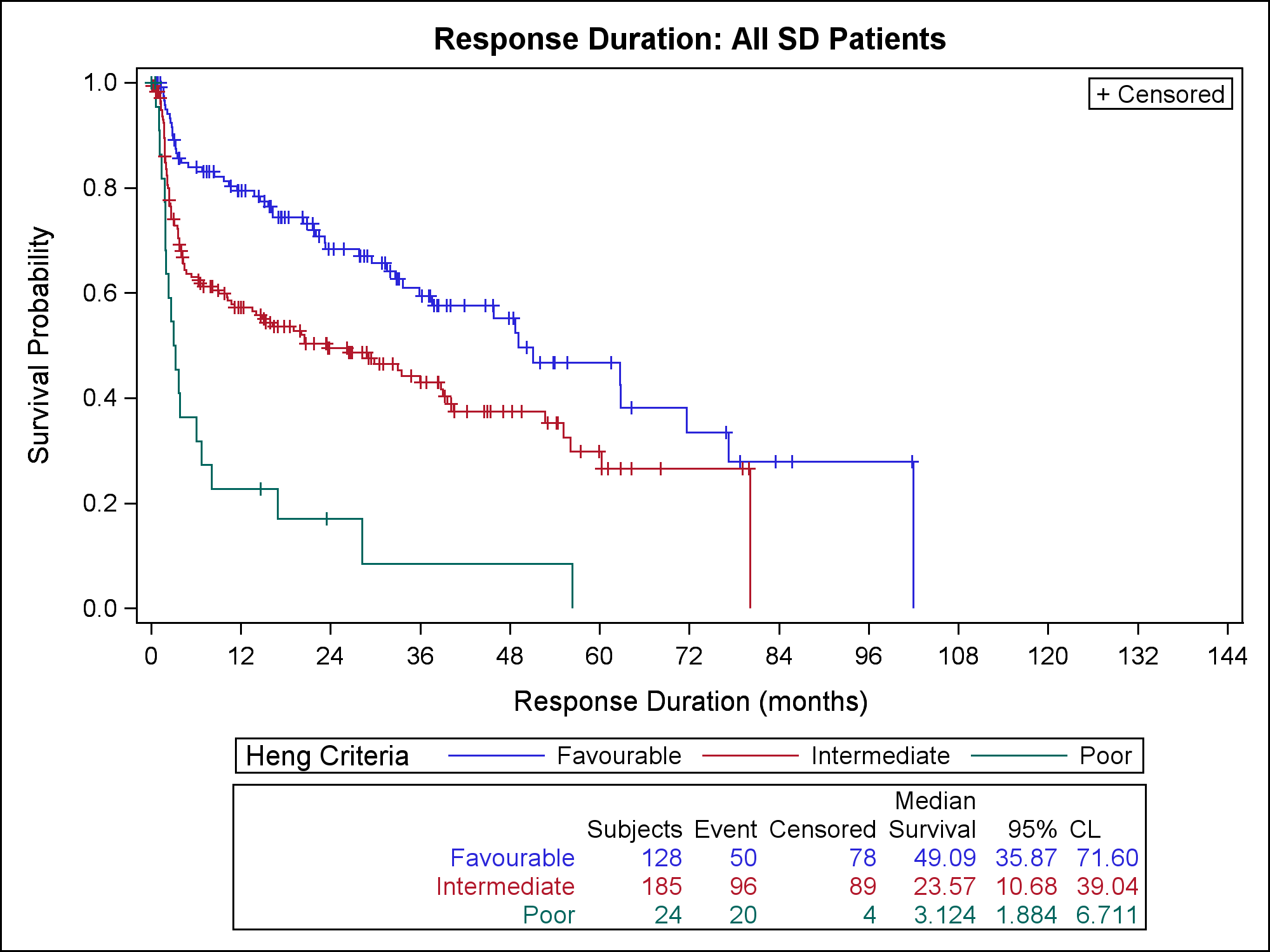


Figure S6: Overall Survival by RCC Risk: Treatment Prior to IL-2 from First Treatment Date


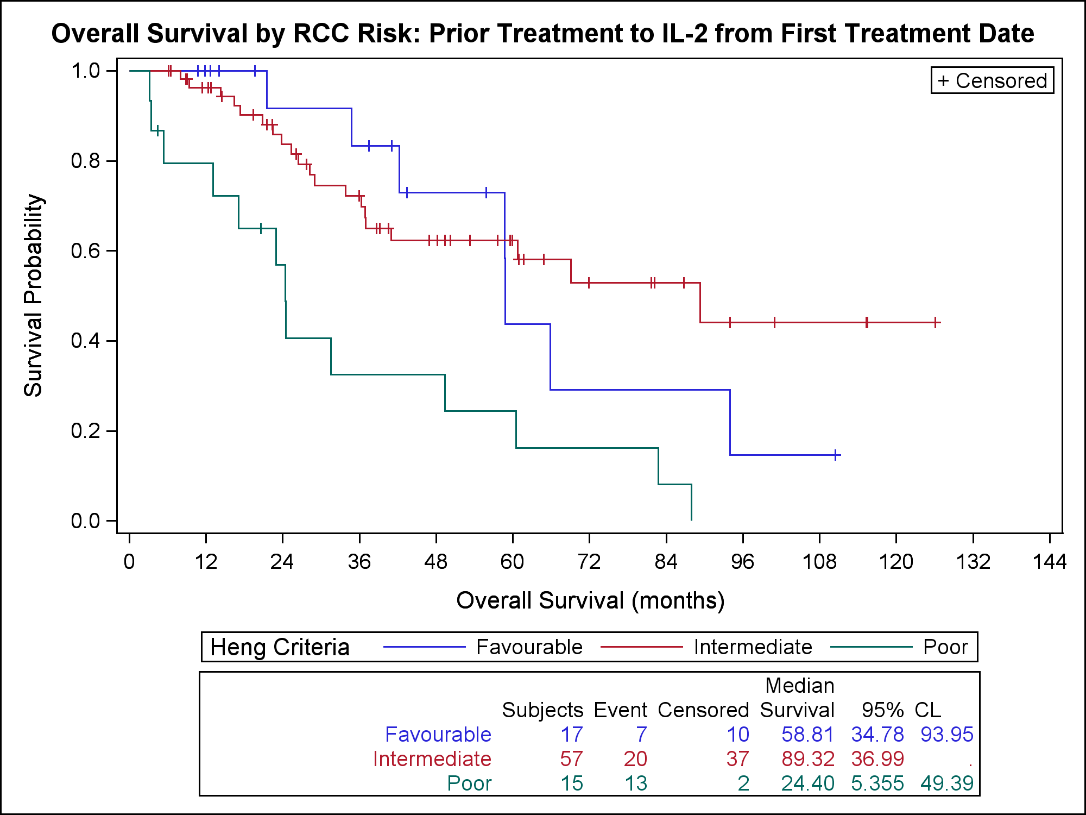

Supplement: Supplementary file 1 — Table S1a. Median survival (months) by risk group and therapy sequence. Table S1b. Two year overall survival by risk group and treatment sequence. Table S2. Ongoing trials of IL-2 and checkpoint inhibitors. Figure S1. Overall survival by RCC risk: all patients with 6 IMDC criteria. Figure S2. Overall survival by RCC risk: post-IL-2 treatment. Figure S3. Response duration: all complete response patients. Figure S4. Response duration: All partial response patients. Figure S5. Response duration: all stable disease patients. Figure S6. Overall survival by RCC risk: treatment prior to IL-2 from first treatment date. (DOCX 549 kb) [file 40425_2019_567_MOESM1_ESM.docx]
